# A “Global–Local” Visual Servo System for Picking Manipulators

**DOI:** 10.3390/s20123366

**Published:** 2020-06-14

**Authors:** Yinggang Shi, Wei Zhang, Zhiwen Li, Yong Wang, Li Liu, Yongjie Cui

**Affiliations:** 1 College of Mechanical and Electronic Engineering, Northwest A&F University, Yangling 712100, China; syg9696@nwsuaf.edu.cn (Y.S.); zx1314@nwafu.edu.cn (W.Z.); lizhiwen@nwafu.edu.cn (Z.L.); yongwang@nwafu.edu.cn (Y.W.); liuli_ren_79@nwsuaf.edu.cn (L.L.); 2 Key Laboratory of Agricultural Internet of Things, Ministry of Agriculture and Rural, Yangling 712100, China; 3 Shaanxi Key Laboratory of Agricultural Information Perception and Intelligent Service, Yangling 712100, China

**Keywords:** tomato picking, visual servo, manipulator, hand–eye coordination

## Abstract

During the process of automated crop picking, the two hand–eye coordination operation systems, namely “eye to hand” and “eye in hand” have their respective advantages and disadvantages. It is challenging to simultaneously consider both the operational accuracy and the speed of a manipulator. In response to this problem, this study constructs a “global–local” visual servo picking system based on a prototype of a picking robot to provide a global field of vision (through binocular vision) and carry out the picking operation using the monocular visual servo. Using tomato picking as an example, experiments were conducted to obtain the accuracies of judgment and range of fruit maturity, and the scenario of fruit-bearing was simulated over an area where the operation was ongoing to examine the rate of success of the system in terms of continuous fruit picking. The results show that the global–local visual servo picking system had an average accuracy of correctly judging fruit maturity of 92.8%, average error of fruit distance measurement in the range 0.485 cm, average time for continuous fruit picking of 20.06 s, and average success rate of picking of 92.45%.

## 1. Introduction

Selective fruit harvesting is among the most time-consuming and labor-intensive agricultural operations. Over the past four decades, humans have been trying to develop robots to do this work [1,2,3,4]. However, owing to the complex operating environment and unstructured physical parameters of the operational objects, several key factors affect the smooth operation of fruit-harvesting robots. One of them is the precise collaborative operation of the target positioning unit of fruit recognition and the picking execution component, also known as the “hand–eye collaborative operation” system [5,6,7].

In the hand–eye coordination operation system of the manipulator, there are two major ways to install cameras, “eye to hand” and “eye in hand” [8]. In the “eye to hand”-based hand–eye coordination operation system, the camera and the manipulator are installed separately, which can help obtain image-related information of the fruit over a larger field of view, where it is easy to realize visual feedback control, but in this system, the movement of the manipulator causes the target object to be occluded. When the environment changes or the camera moves, the latter needs to be recalibrated [9,10]. The accuracy of the visual system and the mechanical system also affect the rate of success of operation, operational accuracy, and cost. In the “eye in hand” hand–eye coordination operation system, the camera is fixed at the end of the manipulator, and the target object is close to it to prevent the manipulator from occluding the target object and to achieve a high-resolution image [11]. The requirements on the accuracies of visual measurement and positioning of the manipulator are low, and the small displacement of the fruit does not affect its grip. However, the system needs to continually call the results of processing of images from the camera because it has a small field of view. Furthermore, using the feedback control of the manipulator, the robot is susceptible to tremors [12], thus influencing the stability of control and picking speed of the system. There is also another method that combines two camera mounting methods, which has been less studied in the field of agriculture. Mehta et al. [13] proposed a cooperative visual servo controller that benefits from the large field-of-view of a fixed camera and the accuracy of a camera-in-hand (CiH). A rotation controller was developed to orient the robot such that the target fruit selected by the fixed camera can be viewed by the CiH attached to the end-effector. At the same time, the corresponding control system was developed and a strict stability analysis is carried out to guarantee its closed-loop performance.

During target identification, positioning, and picking among automated picking operations, if the advantages of the first and second operating systems described earlier can be combined to coordinate the vision system, the movement chassis, and the manipulator system, accurate operation can be achieved. In this paper, our method is similar to the third one mentioned above. A binocular camera was mounted onto the chassis of a picking robot and a monocular camera was mounted at the end of the picking manipulator. A “global–local” visual servo picking system was thus constructed. The binocular camera was used to identify ripe fruits and calibrate the operational distance. The robot then moved to the area of the picking operation and picked fruit using the manipulator with a monocular camera servo. Compared with the first method mentioned above, the visual accuracy and mechanical accuracy of the method proposed in this paper greatly reduced the influence on the accuracy of fruit picking. It also had the advantage of the second method, the smaller displacement of the fruit did not affect the success rate of picking. There were two main differences with the third method mentioned above. Firstly, their overall system was fixed. A fixed monocular camera was used to provide a global view and obtain the target fruit information of the whole fruit tree. However, our overall system is mobile. The fixed binocular camera was used to obtain the information of fruits in the front working area, and meanwhile, the chassis moving platform was coordinated to reach the work stopping point. Secondly, they got the 3D fruit position through the camera in the center of the terminal claw, completing the real-time control of the manipulator. In contrast, we obtained the target fruit image information through the camera in the center of the terminal claw and realized the visual servo control based on the image to complete the harvest. Feng et.al. [4] designed an autonomous tomato harvesting robot and they used the first method of vision servo. The execution time of a single harvest cycle was about 24s, and the success rate for harvesting tomatoes was 83.9%. Lehnert et al. [12] designed an autonomous sweet pepper harvesting system for protected cropping systems and they used the second vision servo method. The highest success rate of grasping was 90%. Mehta et al. [13] proposed a cooperative visual servo controller for harvesting medium and large varieties of citrus fruit. The accuracy of the controller was observed to be about 15 mm. The results of experiments showed that the system we proposed is feasible. Compared with the first two methods mentioned above, this system can perform picking operations highly reliably while improving picking accuracy and speed in automated agricultural picking.

The presented paper is organized as follows: Section 2 introduces the principle of “global–local” visual servo picking and describes the operating process of the system; Section 3 states every subsystem of the system, which includes manipulator workspace analysis, visual processing methods and picking strategies; Section 4 provides experimental evidence to verify the feasibility of this “global–local” visual servo system; concluding remarks are presented in Section 5.

## 2. Principle of “Global–Local” Visual Servo Picking

**Figure 1:**
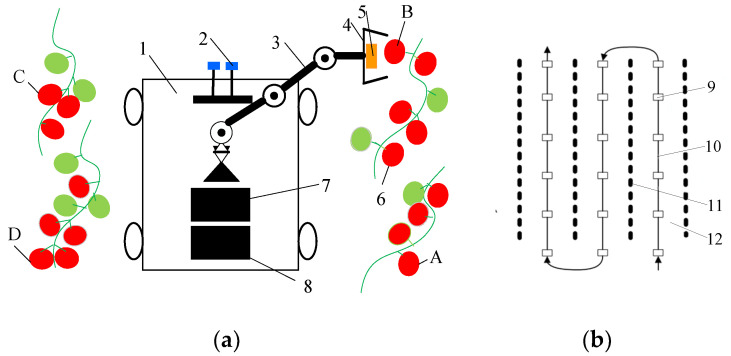
(**a**) “Global–local” visual servo picking system; (**b**) Cruise path of the robot. 1. Chassis; 2. Binocular vision system; 3. Manipulator; 4. End effector; 5. Monocular vision system; 6. Fruits; 7. Manipulator motion controller;8. Chassis motion controller; 9. Stops during robot operation; 10. Cruise path of robot; 11. Tomato-planting ridge; 12. Aisle between ridges.

The “global–local” visual servo picking system, as shown in Figure 1a, includes a monocular vision system, a binocular vision system, a chassis, a manipulator, and an end effector. The robot performs cruise operations according to the specific operational path as shown in Figure 1b. When the robot stops at the first stop point before entering the ridge, the binocular vision system starts to judge the ripeness of the fruit in the global field of vision, and calibrates the operational distance of the ripe fruit at the given coordinates. Then, by reference to the operational space of the manipulator, the range of operations for picking ripe fruits can be determined. Under the guidance of the robot’s navigation and positioning system, the chassis motion system is driven to move to the calculated operational stop point. The manipulator begins picking operations under monocular visual guidance. During the operation of the manipulator, the binocular vision system begins to judge the maturity of the fruit in the global field of vision, and calibrates the distance to the ripe fruit in cycles for automated picking operations.

**Figure 2:**
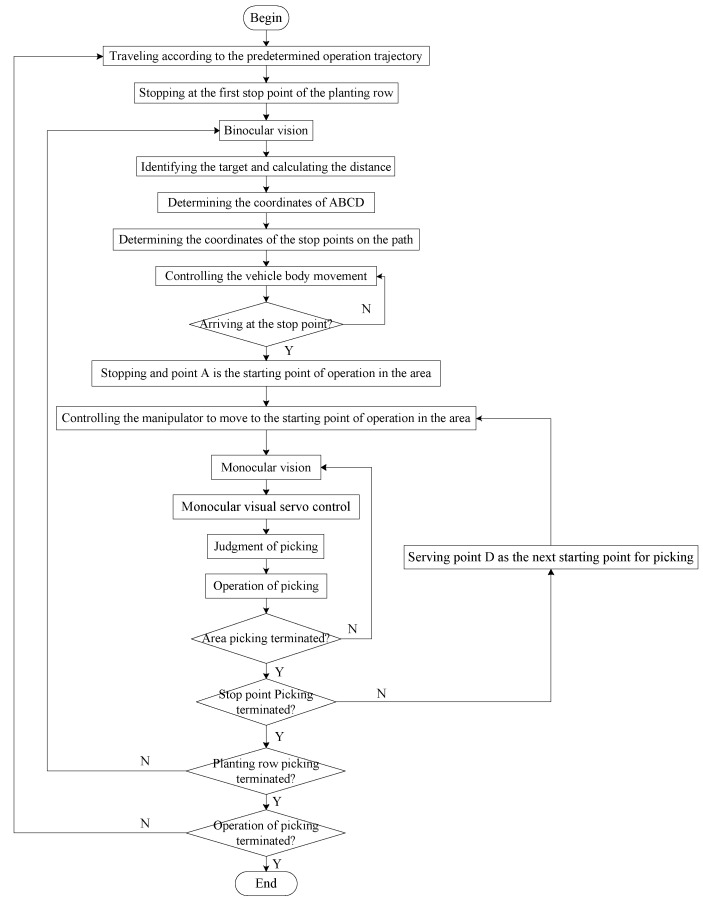
Process of the robot’s visual servo picking operation.

The process of visual servo picking operations is shown in Figure 2. The robot travels according to a predetermined operational trajectory and stops at the first stop point of the ridged aisle. The binocular vision system begins to judge the degree of ripeness of the fruit in the global field of vision and demarcates the operational distance to the ripe fruit at the given coordinates. Based on an analysis of the operating space of the manipulator, the range of operation of the ripe fruit that can be picked is obtained. The coordinates of the next stop point for the robot and the farthest fruits—A, B, C, and D—that can be picked by the robot as well as their respective picking coordinates are planned. Then, under the guidance of the navigation and positioning system, the robot controls the chassis motion system to stay at the predetermined stop points for operation, and drives the end of the manipulator to reach the vicinity of fruit A. By using the monocular vision system, the servo controls the manipulator to move to the picking position for fruit A and starts picking the ripe fruit on the right side of the robot’s forward direction. When the distance between the end effector and the target fruit meets the picking requirements, a single picking operation is complete. Then, the remaining ripe fruits are picked via the monocular visual servo. When the end of the manipulator moves to the vicinity of fruit B, the operation of the picking of the ripe fruit on the right side of the robot is carried out. When the end of the manipulator moves to the vicinity of fruit D, it starts picking the ripe fruit on the left side of the robot. When the end of the manipulator moves to the vicinity of fruit C, the ripe fruit is picked on the left side of the robot. Once all ripe fruits in the area near the robot have been picked, it utilizes the binocular vision system to continue to judge fruit ripeness in the global field of vision and demarcate the operational distance to the ripe fruit. It travels to the next target stop point and repeats the above steps to continue picking the ripe fruit in other areas. Once the picking operation in the given ridge aisle is complete, the robot continues to travel according to its predetermined operating trajectory and proceeds to the next ridge aisle to continue picking.

**Figure 3:**
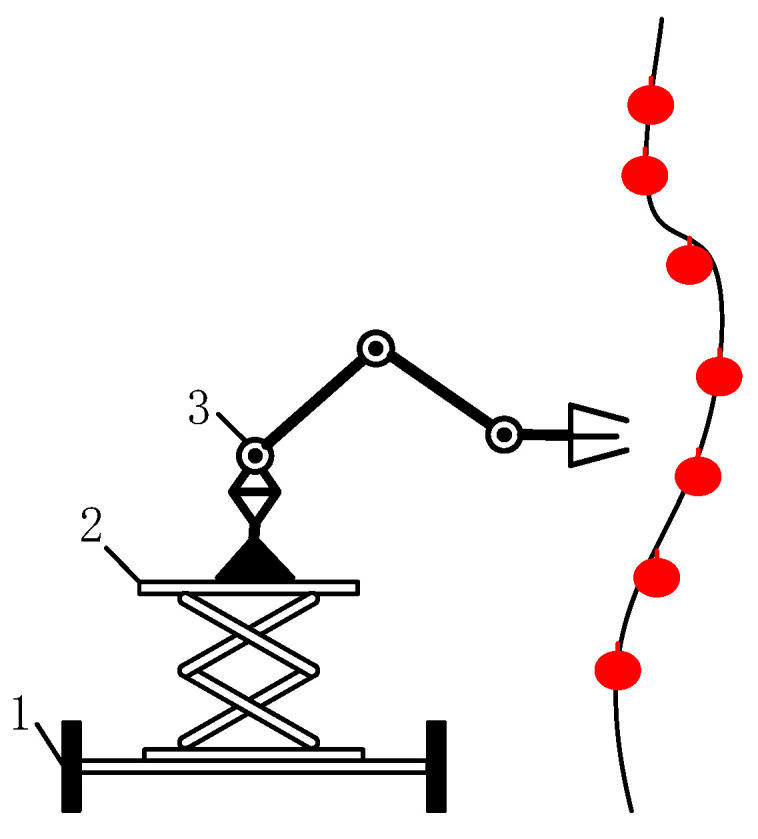
Lifting platform required by robots to pick tomatoes at different stages of maturity. 1. Chassis; 2. Lifting platform; 3. Picking manipulator.

Based on an analysis of the process of the robot’s picking operation, methods for determining the operational space of the manipulator, the range of accuracy of the binocular vision system, the positions of fruits A, B, C, and D, and the determination of the starting points for picking influence the calculation of coordinates of the robot’s stop points. The accuracy of converting the binocular camera’s coordinate system and the manipulator’s end-coordinate system influence and determine the efficiency of the picking operations. The coordinated operation of the monocular vision system, manipulator, and end effector determine the order of picking of the ripe fruit and the timing of the picking action. As different greenhouses have different planting specifications and heights of fruit growth, different robotic picking manipulators are required. To carry out standardized picking operations and improve the efficiency of the automatic operation of the robot, the automatic picking of tomato in a greenhouse was used as an example. The robot requires different manipulators when picking tomatoes at different heights. However, in general, tomatoes close to the root begin to mature first, after which their height continues to increase as they ripen. Moreover, tomatoes at the same height have slight differences in times of maturation. As a result, the lifting platform shown in Figure 3 can be used in combination with a robot’s chassis and a manipulator to pick ripe tomatoes in different periods. In this way, the principles of automatic picking of tomatoes at different heights are roughly the same, with the difference being that as the heights of the tomatoes vary, the adjustments to the height of the platform vary. Therefore, for the picking discussed here, the manipulator can have access to all tomatoes within the field of vision for picking.

**Figure 4:**
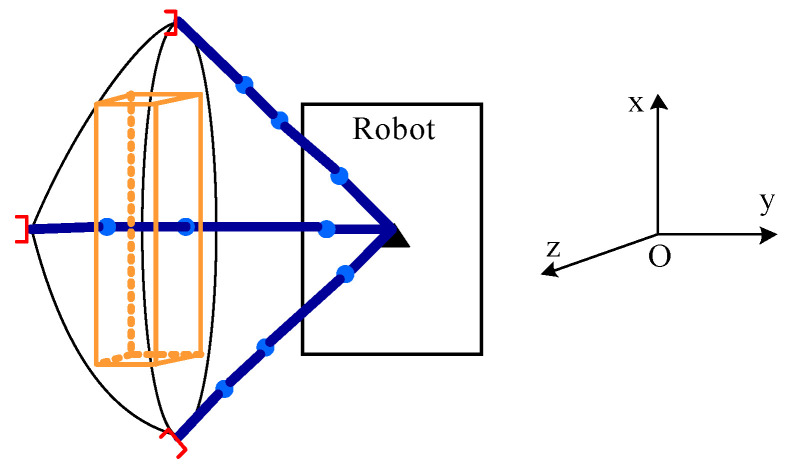
Operational area for automatic picking of tomatoes in the greenhouse.

**Figure 5:**
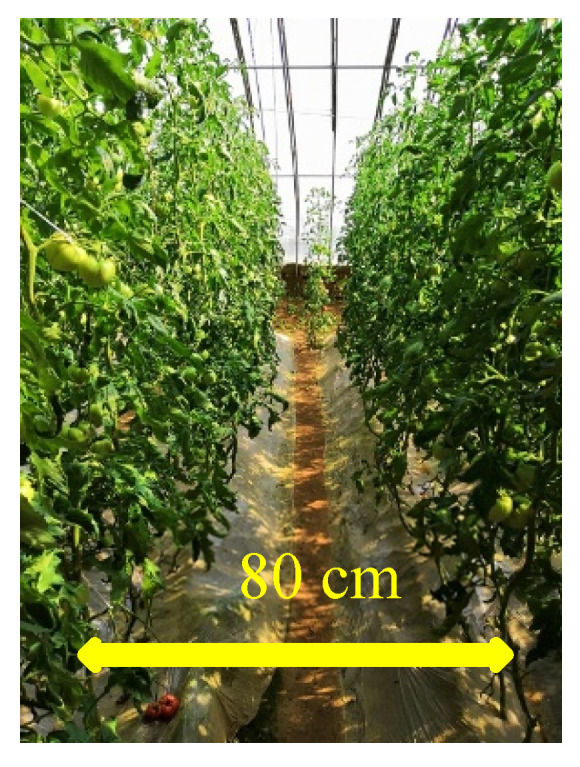
Inter-ridge distance of tomatoes cultivated in the greenhouse.

**Figure 6:**
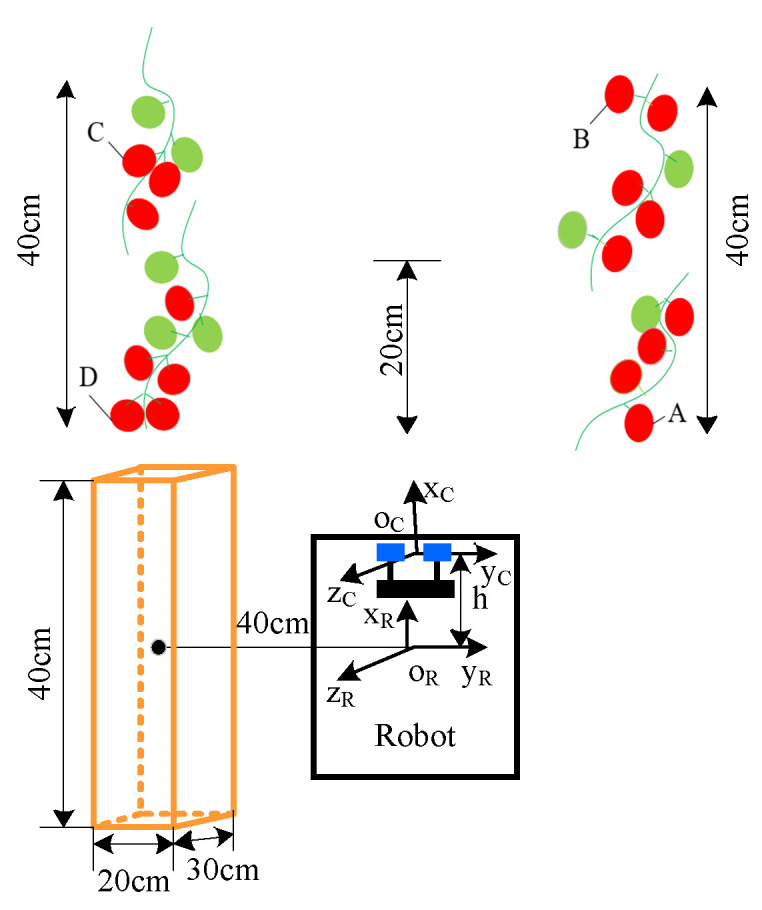
Confirmation of the operational area for picking tomatoes and the next stop point.

Assuming that all joints of the picking manipulator were rotated by 180 degrees and the base joint was rotated, the theoretical operational area of the robot was a round crown shape. As shown in Figure 4, under normal circumstances, tomatoes cultivated in the greenhouse had an inter-ridge interval of 80 cm, thickness of the plant of 15 cm, and an effective plant height of 30~160 cm as shown in Figure 5. To enable the robot to perform picking operations effectively and simultaneously facilitate the continuous planning of its picking trajectory, the picking area was taken as a rectangular parallelepiped as shown in Figure 6. According to the inter-ridge distance for planting tomatoes in the greenhouse and plant thickness, the distance between the origin of the manipulator and the effective area of operation of the robot was set to 30–50 cm; that is, the length of the area of operation shaped as a rectangular parallelepiped along the y-axis was 20 cm, and the length along the x-axis was half the distance of the inter-ridge plant interval, that is, 40 cm. The length along the z-axis was 30 cm.

As shown in Figure 6, the center of symmetry of the two lenses of the binocular camera was taken as the origin of its coordinate system, and the center of the bottom of the base of the manipulator was set as the origin of its coordinate system. The coordinates of the ripe fruit in the field of vision were detected by the binocular vision system, and the distances between each fruit, and the x-, y-, and z-axes of the binocular camera were calculated. According to the positional relationship between the binocular camera and the base of the manipulator, the coordinates of the ripe fruit relative to the axes of the base of the manipulator were determined. In the picking area on the right side of the robot, the ripe fruit at the shortest distance from the binocular vision system along the x-axis was selected and marked as fruit A. In the picking area on the left side of the robot, the ripe fruit at the shortest distance from the binocular vision system along the x-axis was marked as Fruit D. The robot traveled in the middle of the ridge. Considering its navigational and positioning accuracy, its coordinates along the y-axis remained unchanged. According to the distances between fruit A and fruit D, and the x-axis of the base of the manipulator, the smaller value of the two was calculated. This was recorded as the starting point of the next space for the picking operation along the x-axis. Coordinates of the starting point along the x-axis and half of the length of the space for the picking operation—that is, 20 cm—were added to obtain coordinates of the x-axis of the next stop point of the robot. The starting point along the x-axis and 40 cm were added to obtain coordinates of the x-axis of the next terminal point of the picking operation of the robot. According to the distance to the fruit along the z-axis, the spatial height of the fruit suitable for the picking operation was chosen. Then, according to the distribution of fruits on the left and right sides of the robot, the coordinates of the ripe fruits were saved. In the space for the picking operation on the right side, the fruit farthest from the binocular vision system along the x-axis was selected and marked as B. In the picking space on the left side, the fruit farthest from the binocular vision system along the x-axis was selected and marked as fruit C. Then, the robot could quickly reach the next stop point for operation via the navigation and positioning system.

**Figure 7:**
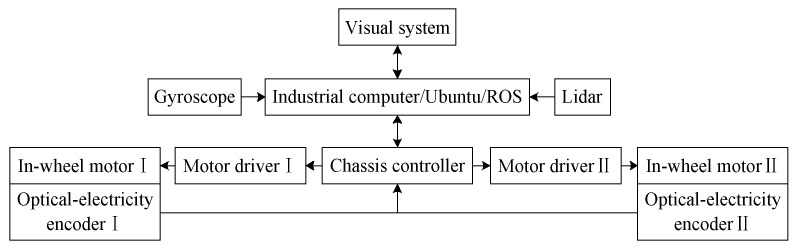
Navigation and positioning system.

For visual servo navigation and positioning, our team used a greenhouse robot that combined a navigation system based on an odometer, a gyroscope, and a laser radar. This could operate while constructing a two-dimensional (2D) environment map for the greenhouse with its system architecture as shown in Figure 7. Its principle was that the robot initially planned a range for the area for the walking operation based on the cruise task of automatic picking. It then obtained mileage information by collecting data from the encoder while walking in combination with data detected by the gyroscope as well as the originally planned walking track through track deduction. The robot established a 2D environment map in combination with data scanned by a laser radar and the gmapping algorithm. The A* algorithm was used to plan the cruise path and the self-adaptive Monte Carlo method (AMCL) to estimate the position and posture of the robot. The application of the A* algorithm was expanded according to the navigation toolkit in ROSA and a specific path of cruise operation was obtained by setting the target points. A considerable amount of research has been devoted to the navigation and positioning of robots [14,15,16,17], and our team is also writing articles on the issue. Accurate navigation and positioning are helpful for automatic picking, but the main purpose of this paper is to introduce the global–local visual servo picking operations of the robot in a greenhouse. Due to limitations of length, details of the robot’s navigation and positioning are provided here.

Based on the above analysis, critical factors influence the performance of the global–local visual servo picking system, including the rate of identification of ripe fruits, the range of accuracy of binocular vision, and the efficiency and success of picking using the monocular visual servo of the manipulator.

## 3. Materials and Methods

### 3.1. Prototype of the Picking Robot

**Figure 8:**
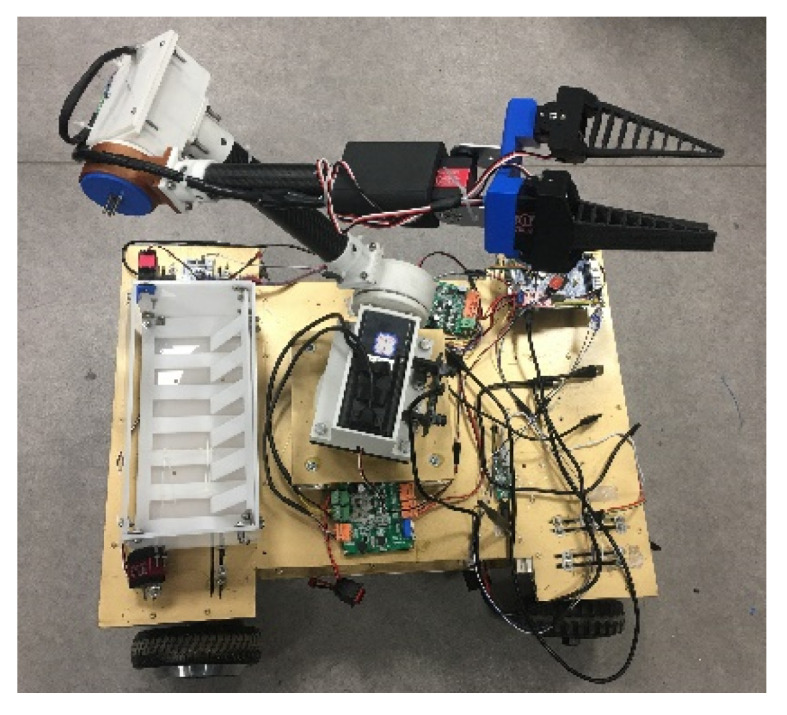
Prototype of the picking robot.

A prototype of the picking robot is shown in Figure 8. It consisted of a wheeled chassis, a binocular camera (3D-1MP02-V92, Rervision Technology Co. Ltd., Shenzhen, Guangdong, China), a four-degree-of-freedom manipulator, an end effector, and controllers. The wheeled chassis was driven by a hub motor (GM37-545, Shenzhen Chihai Electric Motor Co. Ltd., Shenzhen, Guangdong, China), and the motor bracket of each wheel was connected to a shock absorber (HITI-100-20-20-15-2 -M, Hiti Technology Co. Ltd., Wuhan, Hubei, China). When any independently moving wheel bounced during driving, the remaining wheels were not significantly affected, rendering the robot’s motion smoother.

**Figure 9:**
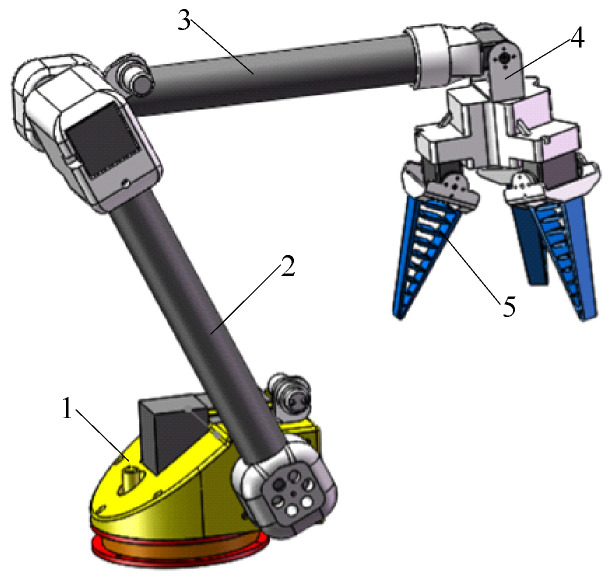
Three-dimensional view of the manipulator. 1. Waist joint; 2. Big arm; 3. Small arm; 4. Wrist joint; 5. End effector.

There were many types of manipulator [18,19,20,21] and the picking robot used a picking manipulator featuring joints with four degrees of freedom as shown in Figure 9. Support for the waist joint motor was equipped with the motor and a shoulder joint motor, which was connected to the large arm of the system. The lumbar joint motor support, connectors at the joints of the large and small arms, and motor support were 3D-printed using Polylactic Acid (PLA) material. The lever of the arms was made of carbon fiber tube (1312, Hong Kong NuKied Industrial Co. Ltd., Hong Kong, China), The length of the lever of the large arm was a2 = 32 mm and that of the small arm was a3 = 29 mm. The wrist joint comprised a U-shaped frame and a two-axis steering gear (RDS3115, Dongguan City Dsservo Technology Co. Ltd., Dongguan, Guangdong, China) made of aluminum, which was interference fitted at the end of the small arm. The distance between the end of the manipulator and the wrist was a4 = 13 mm. The motor for each joint of the manipulator used a DC brushless motor (42BL80S09-230TR9, Time Chaoqun Technology Co. Ltd., Beijing, China), and was equipped with a harmonic reducer (CSF-17-100-2UH, Harmonic Drive Systems Inc, Tokyo, Japan), with a transmission ratio of 1:100.

**Figure 10:**
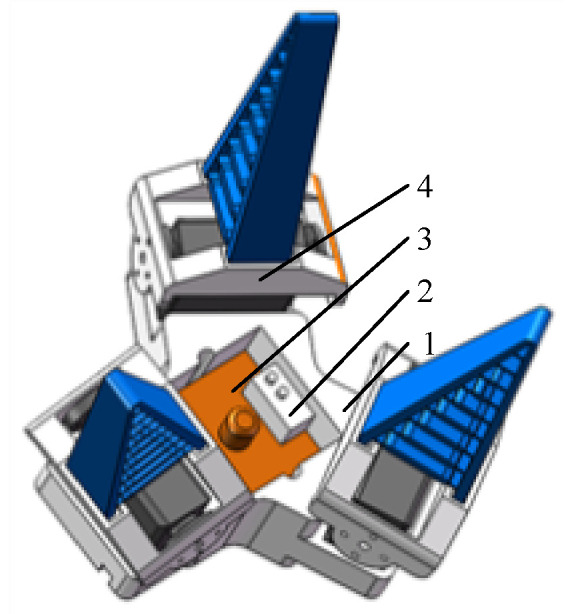
Three-dimensional view of the end effector. 1. Palm; 2. Square photoelectric switch; 3. Monocular camera; 4. Flexible fingers.

The end effector is shown in Figure 10, and included the palm, square photoelectric switch (E3Z-LS61-TB, TAYB Electronic Technology Co. Ltd., Suzhou, Jiangsu, China), monocular camera (QR-USB3MP01H, Rervision Technology Co. Ltd., Shenzhen, Guangdong, China), and flexible fingers. Each flexible finger was composed of a dual-axis steering gear (RDS3115, Dongguan City Dsservo Technology Co. Ltd., Dongguan, Guangdong, China), a fin-bar effector bracket, and a fin-bar effector. The dual-axis steering gear was secured to the effector’s bracket through the steering wheel. A fin-strip effector was fixed in the groove of the bracket. This is a flexible finger designed based on the fin-strip effect [22] and was 3D-printed using flexible TPU material. Brackets of the palm and fin-strip effector were 3D-printed using PLA material.

**Figure 11:**
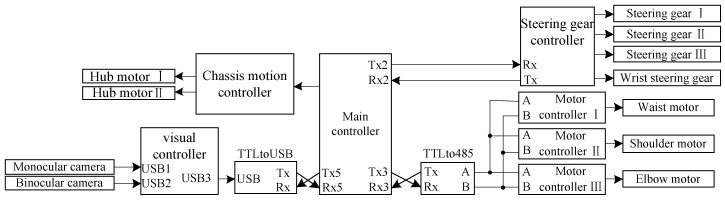
Structure diagram of the control system.

The structure of the control system for visual servo picking is shown in Figure 11. The monocular and binocular cameras were connected to the visual controller (D12120P551, Shenzhen Hongdafeng Electronics Co. Ltd., Shenzhen, Guangdong, China) via a USB interface to form a visual system, and to acquire and process image-related information on the fruit. The main controller (Battleship V3, Xingyi Electronic Technology Co. Ltd., Guangzhou, Guangdong, China) obtained feature information from images processed by the visual controller via serial port 5. The main controller drove each motor of the joints of the picking robot by sending commands to the controller (AQMD3608BLS, Aikong Electronic Technology Co. Ltd., Chengdu, Szechwan, China) via serial port 3. The main controller was connected to the steering gear controller (LSC-16-V1.3, Shenzhen Hiwonder Technology Co. Ltd., Shenzhen, Guangdong, China) via serial port 2 to control the movement of the steering gear of the joint of the wrist and fingers of the end effector. The main controller was connected to the chassis motion controller (DC-30A, Shenzhen Britt Technology Co. Ltd., Shenzhen, Guangdong, China) through several pins to drive the hub motor of the chassis.

### 3.2. Analysis of the Manipulator’s Operation Space

**Figure 12:**
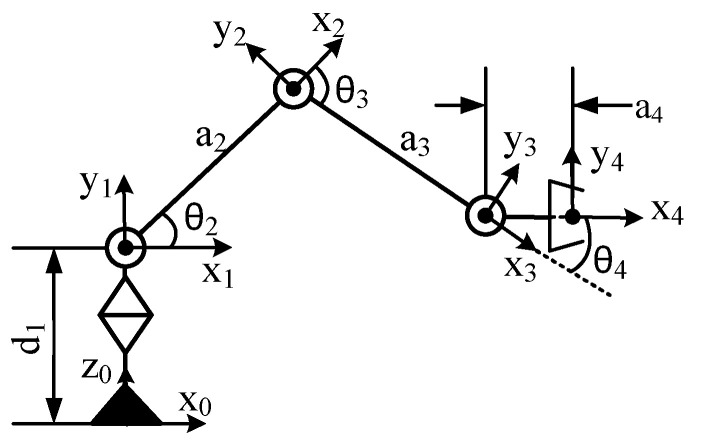
Coordinate system of the manipulator.

The D-H method was used to establish the coordinate system of the picking manipulator as shown in Figure 12, with parameters as shown in Table 1. Coordinate system {0} was the base coordinate system of the manipulator and coordinate system {4} was its end coordinate system. The transformation matrix connecting rod i-1 was the transformation matrix for coordinate system {*i*} to coordinate system {*i*-1}

(1) $$A_{i}^{i-1}=\left[\begin{array}{cccc} c\theta_{i} & -s\theta_{i}c\alpha_{i} & s\theta_{i}s\alpha_{i} & a_{i}c\theta_{i}\\ s\theta_{i} & c\theta_{i}c\alpha_{i} & -c\theta_{i}s\alpha_{i} & a_{i}s\theta_{i}\\ 0 & s\alpha_{i}^{} & c\alpha_{i}^{} & d_{i}\\ 0 & 0 & 0 & 1 \end{array}\right]$$

The transformation matrices of connecting rods 1, 2, 3, and 4 are:

(2) $$A_{1}^{0}=\left[\begin{array}{cccc} c_{1} & 0 & s_{1} & 0\\ s_{1} & 0 & -c_{1} & 0\\ 0 & 1 & 0 & d_{1}\\ 0 & 0 & 0 & 1 \end{array}\right],A_{2}^{1}=\left[\begin{array}{cccc} c_{2} & -s_{2} & 0 & a_{2}c_{2}\\ s_{2} & c_{2} & 0 & a_{2}s_{2}\\ 0 & 0 & 1 & 0\\ 0 & 0 & 0 & 1 \end{array}\right],A_{3}^{2}=\left[\begin{array}{cccc} c_{3} & -s_{3} & 0 & a_{3}c_{3}\\ s_{3} & c_{3} & 0 & a_{3}s_{3}\\ 0 & 0 & 1 & 0\\ 0 & 0 & 0 & 1 \end{array}\right],A_{4}^{3}=\left[\begin{array}{cccc} c_{4} & -s_{4} & 0 & a_{4}c_{4}\\ s_{4} & c_{4} & 0 & a_{4}s_{4}\\ 0 & 0 & 1 & 0\\ 0 & 0 & 0 & 1 \end{array}\right]$$

$s_{i\cdots j}$ represents $\sin(\theta_{i}+\cdots +\theta_{j})$, and $c_{i\cdots j}$ represents. The equation for the transformation of the coordinate system {4} of the end of the manipulator to the base coordinate system {0} was:

(3) $$A_{4}^{0}=A_{1}^{0}A_{2}^{1}A_{3}^{2}A_{4}^{3}=\left[\begin{array}{cccc} c_{1}c_{234} & -c_{1}s_{234} & s_{1} & c_{1}(a_{2}c_{2}+a_{3}c_{23}+a_{4}c_{234})\\ s_{1}c_{234} & -s_{1}s_{234} & -c_{1} & s_{1}(a_{2}s_{2}+a_{3}s_{23}+a_{4}s_{234})\\ s_{234} & c_{234} & 0 & a_{2}s_{2}+a_{3}s_{23}+a_{4}s_{234}+d_{1}\\ 0 & 0 & 0 & 1 \end{array}\right]$$

As shown in Figure 1, the binocular camera was located directly in front of the base of the manipulator at a distance of h = 20 cm. The base coordinate system of the manipulator {0} did not undergo a posture transformation relative to the coordinate system of the binocular camera C_1_, and only positional transformation occurred. Accordingly, the equation for the transformation of the base coordinate system {0} of the manipulator relative to that of the binocular camera C_1_ is:

(4) $$A_{0}^{C_{1}}=\left[\begin{array}{cccc} 1 & 0 & 0 & -h\\ 0 & 1 & 0 & 0\\ 0 & 0 & 1 & 0\\ 0 & 0 & 0 & 1 \end{array}\right]$$

Combining Equations (3) and (4), the coordinate system of the end of the manipulator could be transformed into that of the binocular camera. The transformation equation is:

(5) $$A_{C_{2}}^{C_{1}}=A_{0}^{C_{1}}A_{C_{2}}^{0}=\left[\begin{array}{cccc} c_{1}c_{234} & -c_{1}s_{234} & s_{1} & c_{1}(a_{2}c_{2}+a_{3}c_{23}+a_{4}c_{234})-20\\ s_{1}c_{234} & -s_{1}s_{234} & -c_{1} & s_{1}(a_{2}s_{2}+a_{3}s_{23}+a_{4}s_{234})\\ s_{234} & c_{234} & 0 & a_{2}s_{2}+a_{3}s_{23}+a_{4}s_{234}+d_{1}\\ 0 & 0 & 0 & 1 \end{array}\right]$$

The Monte Carlo method [23,24] was used in conjunction with the length of the large and small arms of the manipulator to solve for its operational space, and the distance best suitable for the picking operation was then analyzed. The operational space of the manipulator was the set of all the points that the end of the manipulator could reach in the base coordinate system space. The homogeneous coordinates of the end point of the manipulator in the coordinate system {4} was expressed as ${\left[0\;0\;0\;1\right]}^{T}$. Then, the coordinates of the point in the base coordinate system are expressed as:

(6) $$\left[\begin{array}{l} {}_{}^{0}x\\ {}_{}^{0}y\\ {}_{}^{0}z\\ 1 \end{array}\right]=A_{4}^{0}\left[\begin{array}{l} 0\\ 0\\ 0\\ 1 \end{array}\right]=\left[\begin{array}{c} c_{1}(a_{2}c_{2}+a_{3}c_{23}+a_{4}c_{234})\\ s_{1}(a_{2}s_{2}+a_{3}s_{23}+a_{4}s_{234})\\ a_{2}s_{2}+a_{3}s_{23}+a_{4}s_{234}+d_{1}\\ 1 \end{array}\right]$$

**Figure 13:**
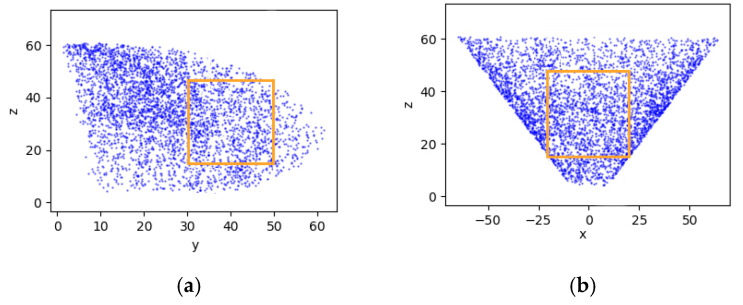

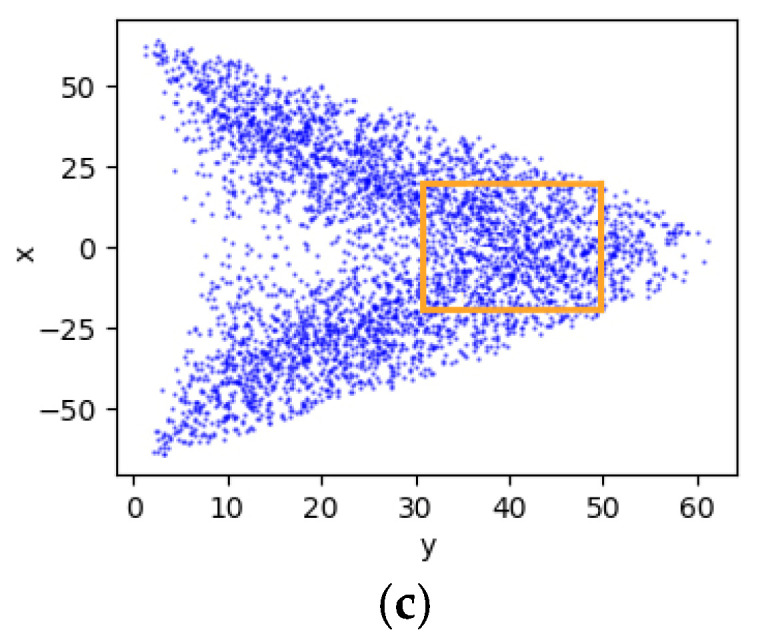
Operational space of the manipulator; (**a**) View along the x-axis; (**b**) View along the y-axis; (**c**) View along the z-axis.

According to the range of the rotation angle of each joint of the manipulator, and in combination with the length and width of the operational space described above, the rand function in Numpy was used to randomly generate 4,000 values within this range. This was substituted into Equation (6) to obtain 4,000 points, and the scatter function in matplotlib was then used to draw these points as the operational space of the manipulator, as shown in Figure 13.

According to the 3D views of the operational space of the manipulator and the rectangular parallelepiped area of operation proposed above, its length along the y-axis was 20 cm, that along the x-axis was 40 cm, and the length along the z-axis was 30 cm, where this could cover many points. The distance between the target fruit within the area of operation and the base of the manipulator met the requirements of Equation (7):

(7) $$\{\begin{array}{c} -20\;\mathrm{cm}\leq x\leq 20\;\mathrm{cm}\\ \;30\;\mathrm{cm}\leq y\leq 50\;\mathrm{cm}\\ \;15\;\mathrm{cm}\leq z\leq 45\;\mathrm{cm} \end{array}$$

According to the parameters of the picking manipulator, a simulation analysis of the operational space was conducted to verify the reasonableness of the chosen area of the picking operation, to determine the range of the distance between the target fruit within the area of operation and the base of the manipulator.

### 3.3. Binocular Identification of Mature Fruits and its Range

The color HSV model used the three parameters of hue H, saturation S, and lightness V to describe color [25]. They were visually independent of one another, and the spatial distance also conformed to the visual characteristics of the human eye. Under certain circumstances, S and V were stable, and H could then reflect the color-related characteristics of the fruit [26]. We used mature tomatoes as an example and judged their maturity by analyzing their color based on the hue.

**Figure 14:**
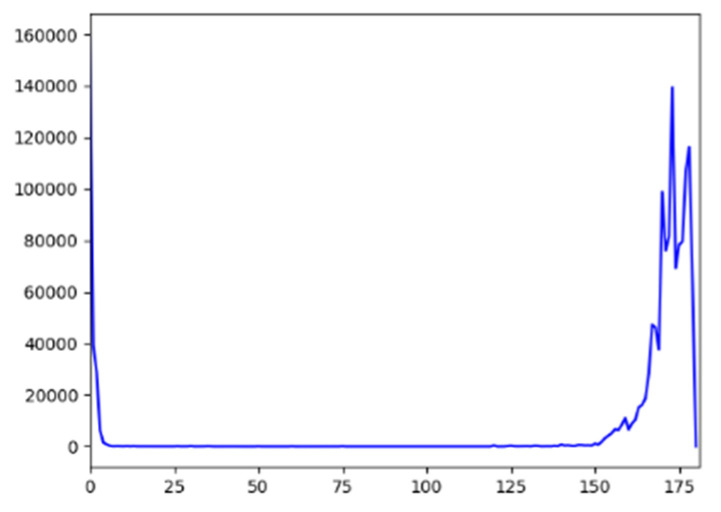
H-channel histogram of area occupied by tomatoes.

The range of the value of hue in the open-source visual library OpenCV was 0–180. A histogram for the statistical analysis of the surface color of mature tomatoes was drawn, with the results shown in Figure 14. It is clear that the hue of mature tomatoes was concentrated between 160 and 180. Considering the significant differences in hue among tomatoes at different levels of maturity, ripe ones could be identified by defining a range of threshold of hue.

**Figure 15:**
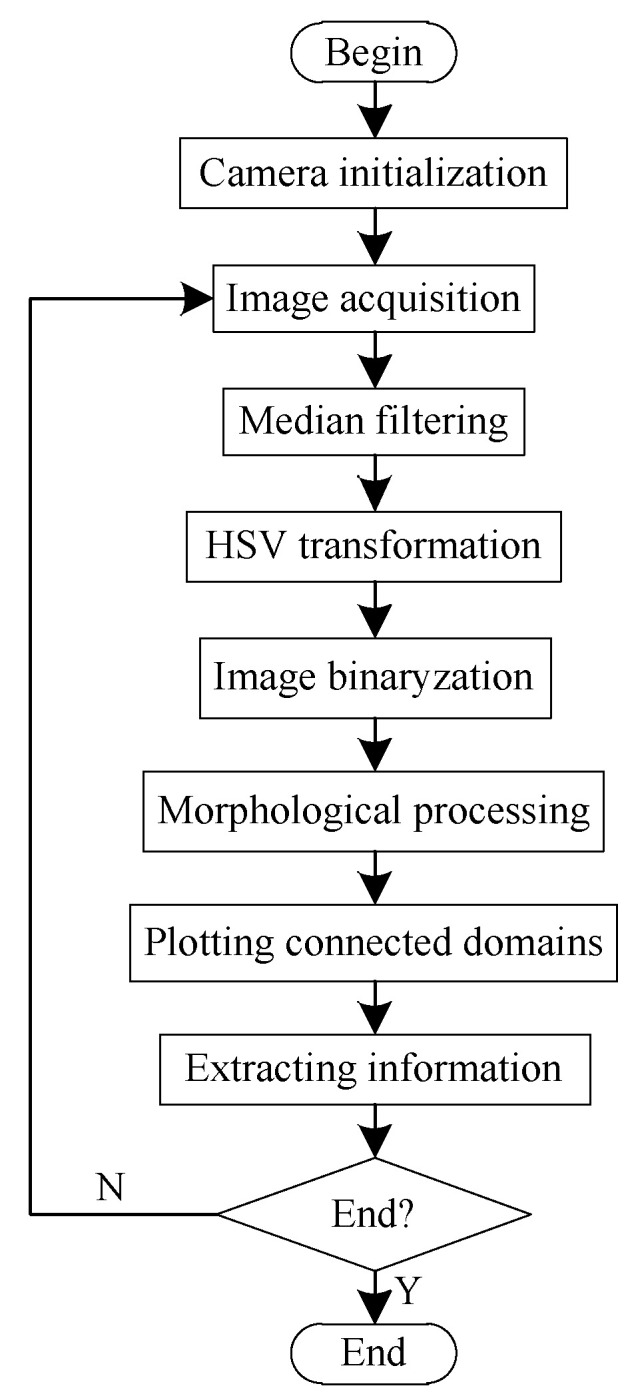
Process for judging ripeness of fruit.

The process of binocular identification is shown in Figure 15. The original image of the fruit was obtained through a binocular camera. Median filtering was used to reduce noise-related interference in the image. The RGB image was then transformed into an HSV image. According to the interval of the values of the hue of mature tomatoes, the image was binarized and morphologically processed to eliminate small white and black areas from it. Finally, all connected domains in the image were identified to identify ripe fruits.

The binocular ranging is a non-contact measurement based on parallax. According to the principle underlying it [27,28,29], as long as the camera’s parameters, and coordinates of the centroid of contours of the fruit in the left and right images can be obtained, ranging can be performed. This requires that the camera be calibrated to establish the relationship between pixels of the image and the position of the scene point. The known image coordinates and world coordinates were employed to obtain the imaging parameters of the camera. Zhang Zhengyou’s calibration method that was used to complete the single-target calibration and stereo calibration of the camera. Parameters of the left and right images of the camera as obtained by single-target calibration are shown in Table 2.

In Table 2, $f_{x},f_{y}$ denote the focal distance of the camera, and P_1_, P_2_, K_1_, K_2_, and K_3_ are the distortion parameters of the lens representing the degree of distortion of the image by it. Using stereo calibration, the relative positional and rotational relationships of the left and right cameras were obtained, as shown in Equation (8), and the translation relationship is shown in Equation (9):

(8) $$R=\left[\begin{array}{ccc} 0.9999 & -6.6466 & -0.0105\\ 6.9344 & 1.0000 & 0.0027\\ 0.0105 & -0.0027 & 0.9999 \end{array}\right]$$

(9) $$T={\left[\begin{array}{ccc} 62.3755 & -0.0383 & -1.5865 \end{array}\right]}^{T}$$

**Figure 16:**
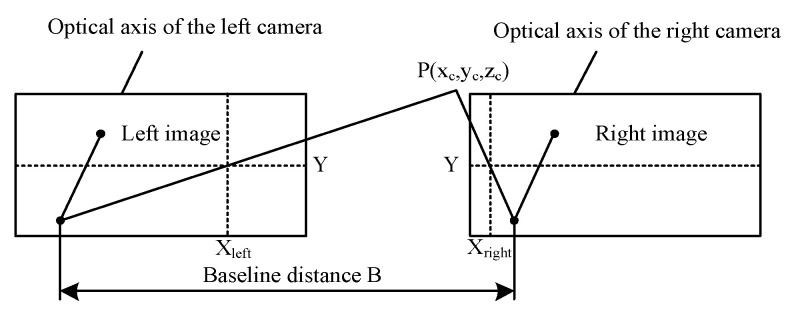
Principle of binocular vision.

The principle of binocular vision is to use two cameras to acquire an image of a 3D spatial point from two angles. The coordinates of the space of the arbitrary object point in binocular field was reconstructed based on the geometric relationship between the corresponding image points in the two images. The binocular vision system was composed of cameras L and R. The pixel size of the camera used was 3.75 μm×3.75 μm and the focal length was $f=1198.9821\times 3.75\times 10^{-3}\;\mathrm{mm}=4.5\;\mathrm{mm}$. The line between the centers of focus is known as the baseline, with length B = 62.4 mm, and the two image planes were in the same plane. The principle of binocular vision is as shown in Figure 16. Two cameras simultaneously photographed the same feature point P of the object space, and coordinates of points P of the left and right eyes were $P_{\mathrm{left}}(X_{\mathrm{left}},Y_{\mathrm{left}})$ and $P_{\mathrm{right}}(X_{\mathrm{right}},Y_{\mathrm{right}})$, respectively. For the feature point of an object, the positions of the image points in the two imaging planes were different. Once the two imaging planes overlapped, the distance between the image points was called parallax.

The images of the two cameras were in the same plane. Thus, the Y coordinates of the point P were identical, i.e., $Y_{left}=Y_{right}=Y$. As a result, the parallax was $d=X_{left}-X_{right}$. According to the geometric relationship, the distance between point P and the baseline was calculated. The greater the parallax was, the closer the scene was to the lens. If P tended to be infinite, the difference would have been approximately zero. For any point on the image on the right side, if there was a corresponding matching point on the image on the left side, the 3D coordinates of the point were determined by seeking geometric solutions, which can be expressed as:

(10) $$\begin{array}{l} \{\begin{array}{c} x_{C}=\frac{B\cdot X_{left}}{d}=\frac{62.4X_{left}}{X_{left}-X_{right}}\\ y_{C}=\frac{B\cdot Y}{d}=\frac{62.4Y}{X_{left}-X_{right}}\\ z_{C}=\frac{B\cdot f}{d}=\frac{280.8}{X_{left}-X_{right}} \end{array} \end{array}$$

### 3.4. Monocular Visual Servo

**Figure 17:**
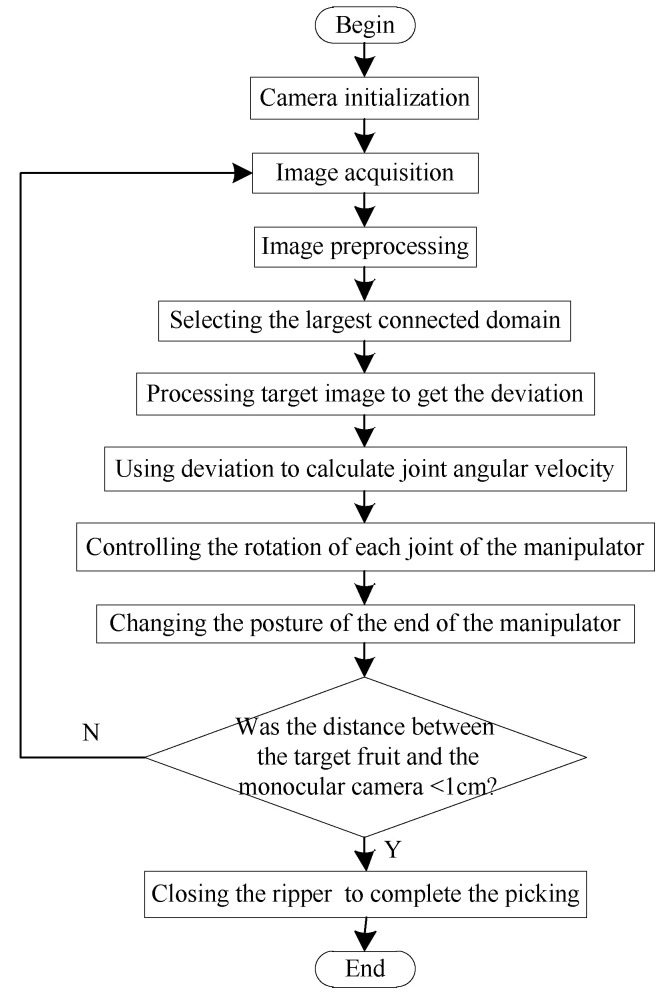
Process of monocular visual servo.

The process of the monocular visual servo approaching the target is shown in Figure 17. The monocular camera located in the center of the gripper took an image and sent it to the visual processor, which performed such preprocessing as filtering on the image (the algorithm for the identification of mature fruit was the same as the binocular identification above). The most connected domain was selected as the image of the target fruit. The horizontal and vertical deviations between the center of the target image and that of the image plane, and the area of deviation were sent to the main controller. Then, the main controller transmitted the output (angular velocity of each joint of the manipulator) calculated by the hand–eye coordination program embedded within it to the motor controller to control the motion of the manipulator. According to the information from the vision processor, the hand–eye coordination program was used to configure the speed register of the motor controller and control the motor in a speed closed-loop manner. The motion of the manipulator, in turn, caused the image in the camera to change, forming a controllable closed loop to achieve the target fruit tracking without processing fruit depth information. A square photoelectric switch was installed in the center of the gripper and its distance was set to l = 1 cm. When the distance between the gripper and the target fruit was short enough, the photoelectric switch was triggered and the gripper was closed to complete the picking operation.

**Figure 18:**
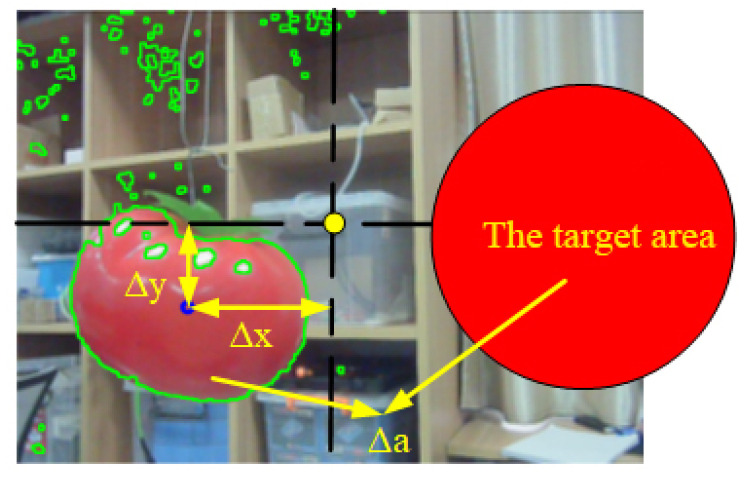
Schematic diagram of the return deviation of the target identification system.

The rotation of joints of the shoulder and elbow of the manipulator influenced the longitudinal deviation and deviation in the distance of the object in the pixel coordinate system, respectively. The rotation of the joint of the waist of the manipulator influenced the lateral deviation of the object in the pixel coordinate system. It was assumed that the longitudinal deviation of the target in the pixel coordinate system changed only due the rotation of the elbow joint, lateral deviation was assumed to change only with the rotation of the waist joint, and the deviation in area was assumed to change only by the rotation of the shoulder joint. The rotation of each joint of the manipulator was controlled by the P algorithm, so that the sum of the three deviations was approximately zero. When the speeds of adjustments of the lateral and longitudinal deviations were faster than that of the deviation in area, the sum of the three deviations could still approach zero quickly and the desired visual servo approach could be achieved. Each deviation is shown in Figure 18.

### 3.5. Experimental Methods

**Figure 19:**
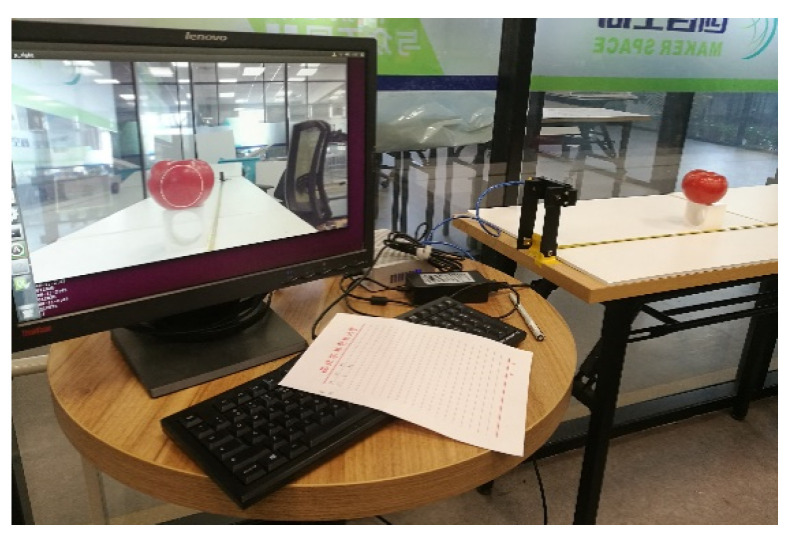
Experimental environment I.

Under a stable light source, a simple binocular vision system was built as shown in Figure 19. To determine the accuracy of the system in terms of judging the maturity of the fruit, tomatoes at different degrees of ripeness were repeatedly and randomly placed within a certain range of distance, and the number of successful identifications were recorded. In addition, the binocular vision system needed to range the ripe fruits. To analyze the accuracy of system ranging, ripe tomatoes were placed at different distances in sequence in the same environment. The preliminarily results were compared with the theoretical values. Rules of the ranging error of the binocular system with regard to distance were then explored. Once the error had been corrected, the ranging experiment was repeated and the results were analyzed to obtain the ranging accuracy of the binocular vision system.

**Figure 20:**
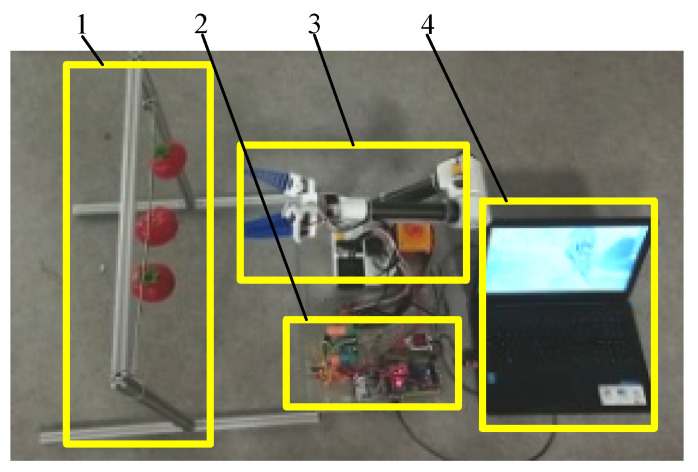
Experimental environment II. 1. Tomato hanger; 2. Control platform; 3. Manipulator; 4. Visual processing platform.

To verify the feasibility of the picking strategy of the monocular visual servo proposed in the paper and analyze its success rate, an experimental platform of monocular visual servo picking was established, as shown in Figure 20. To display the monocular images, we used a personal computer as visual processing platform. The simulated tomato fruit was placed in a random position on the hanger for the manipulator to pick. The image after each visual processing was saved. Multiple images in the picking process at equal intervals were extracted and combined into one image. The trend of variation in the images was observed to verify the feasibility of the monocular visual servo picking strategy.

Before conducting the experiment to analyze the rate of success at picking, the rectangular parallelepiped area for the picking operation described above was verified and the simulated fruits were placed at its eight vertices. The picking of the tomato at each position was repeated several times and the rates of success were recorded. Following this, multiple simulated fruits with different degrees of ripeness in different positions were hung on the shelf at the same time to simulate the distribution of partial fruits in an area. The monocular vision system identified and controlled the tomatoes in the gripper one by one. Multiple experiments were carried out by changing the heights and order of arrangement of the fruits. The time points of gripping were recorded to examine the success rate of the robot for continual fruit picking.

## 4. Experimental Results and Analysis

### 4.1. Efficiency of Discrimination of Fruit Maturity

A total of 120 tomatoes were used, consisting of 83 mature and 37 immature ones. Under stable lighting, the tomatoes were randomly placed within 20 cm–130 cm of the camera, and their maturity was determined using binocular vision. The experiment was repeated three times and the results are shown in Table 3. The average accuracy rate was 92.8%, indicating that the binocular vision system accurately judges the maturity of the tomatoes.

### 4.2. Binocular Ranging Accuracy

**Figure 21:**
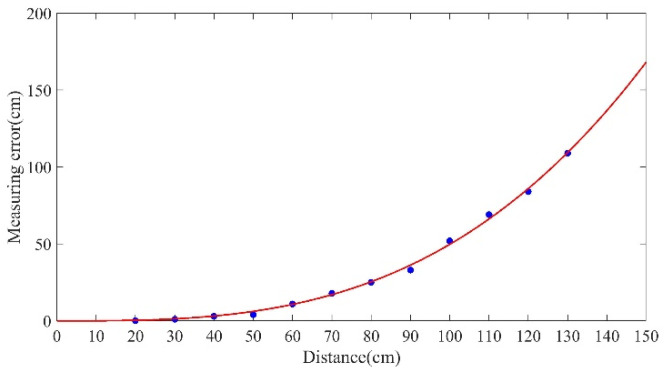
Fitting results.

The distance between the tomatoes and the camera was measured with the binocular system, at intervals of 10 cm over a distance of 20 cm to 130 cm. The measurement errors corresponding to the different distances obtained from the experiment were imported into MATLAB for curve-fitting. The curve is shown in Figure 21. The relationship number of the fitting curve R-square was = 0.9977. It is clear from the figure that the distance-related error was within the allowable range of 20 cm–50 cm. Starting at 60 cm, the error gradually increased, and became larger as the distance increased. In the experiment, due to the effects of light, the resolution of the camera, the processing algorithm, and other conditions, the calculated normalized center moment did not completely coincide with the actual centroid of the object. Thus, the longer the distance was, the larger the error was, as shown in Figure 21.

This error needed to be corrected. Equation (10) for the fitting curve was used to calculate the error corresponding to different distances measured between the camera and the tomato. The distance calculated by this formula minus this error yielded more accurate distance:

(11) $$f(x)=4.884\times 10^{-5}x^{3.004}$$

**Figure 22:**
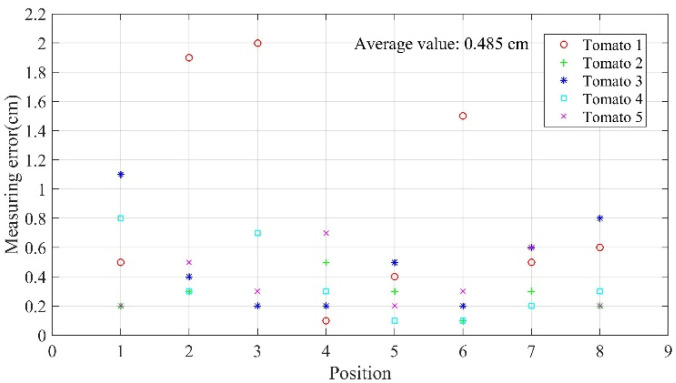
Scatter diagram of errors in distance measurement of tomatoes at different locations.

Five tomatoes were randomly selected, and experiments were conducted under good lighting to verify the accuracy of the modified system. Each tomato was set at eight different locations, and 40 sets of valid data were thus obtained through the experiment as shown in Figure 22. It is clear that the maximum ranging error of the system was 2 cm, average ranging error was 0.485 cm, 90% of the ranging error was smaller than 1 cm, and 72.5% of the ranging error was within 0.6 cm.

### 4.3. Feasibility of Monocular Visual Servo Picking

**Figure 23:**
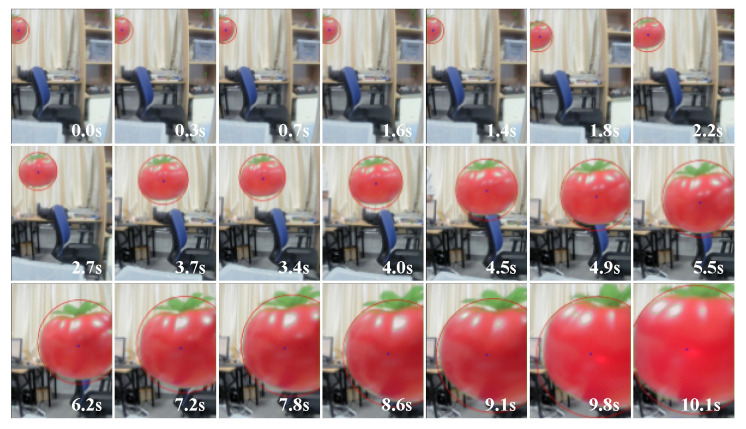
Camera taking images when the manipulator approached the target.

A simulated tomato fruit was placed in a random position on a shelf, and the manipulator was controlled to pick it. The image after each instance of visual processing was stored, and 21 images in the picking process were extracted at equal intervals and combined, as shown in Figure 23. It is clear that the tomato to be picked was in the middle of the image, and the manipulator was consistently able to approach it. Therefore, the monocular visual servo picking strategy proposed here is feasible.

### 4.4. Success Rate of Fruit Picking

The picking space designed for the manipulator was a 3D rectangular parallelepiped area. The tomatoes were placed at the eight vertices of this area and each was gripped by the manipulator 50 times. The number of successful attempts and the time consumed were recorded. The operational area of the manipulator was thus verified. The experimental data are shown in Table 4. It is clear that the manipulator successfully picked the tomatoes at the eight vertices, with an average success rate of 91.5%. Thus, the rectangular parallelepiped working area for picking proposed above is reasonable.

**Figure 24:**
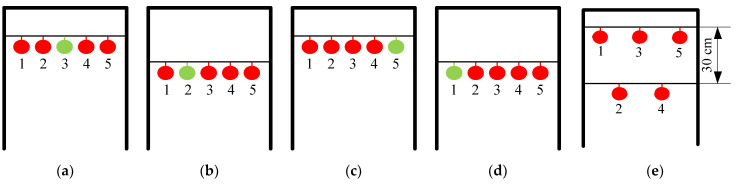
(**a**) Schematic diagram of Fruit hanging I; (**b**) Schematic diagram of Fruit hanging II; (**c**) Schematic diagram of Fruit hanging III; (**d**) Schematic diagram of Fruit hanging IV; (**e**) Schematic diagram of Fruit hanging V.

Five tomatoes were hung at equal intervals on a simulated fruit placement rack, and one of them was a green, immature tomato. Four sets of experiments were carried out and each was repeated 50 times. The manner of hanging the fruit is shown in Figure 24a,b,c,d and the experimental data are shown in Table 5. We also experimented by changing the height and distance between the fruits, as shown in Figure 24e. The average success rate of manipulator in picking the red tomatoes was 92.45%, and the green tomato was never picked. The average time taken for picking a single fruit was 20.06 s.

## 5. Conclusions

The “global–local” visual servo system of the picking manipulator proposed in the paper provides a global field of vision through binocular vision and implements fruit picking using the monocular visual servo. This system can help reduce the impact of the accuracies of visual measurement and the manipulator’s positioning on the success rate of picking operations. At the same time, small displacement of the fruit does not affect gripping it. The system can improve control stability and picking speed in agricultural automated picking. Due to limitations of time and experimental equipment, the authors did not examine optimizing the picking paths of the manipulator. In future work, we will study strategies of picking operations to optimize them. And the visual processing algorithm will be further studied to reduce the impact of unstable illumination on the system accuracy and improve the system’s ability to process visual information.

## Figures and Tables

**Table 1:** D-H parameter table of the manipulator.

Rod	a_i_	α_i_/ (°)	di	θ_i_/ (°)	Range of Variables
1	0	90	d1	θ_1_	−80~80
2	a_2_	0	0	θ_2_	50~130
3	a_3_	0	0	θ_3_	−60~−130
4	a_4_	0	0	θ_4_	−80~80

**Table 2:** Results of camera calibration.

Parameter	Left Camera	Right Camera
f_x_	1198.9821	1201.4563
f_y_	1197.5947	1200.3621
P_1_	0.1021	0.1176
P_2_	−0.2750	−0.3068
K_1_	0.0016	0.0020
K_2_	0.0016	0.0034
K_3_	0	0

**Table 3:** Results of maturity discrimination.

Time	Ripe Tomatoes	Immature Tomatoes	Errors	Accuracy
1	80	32	8	93.3%
2	78	33	9	92.5%
3	77	34	9	92.5%

**Table 4:** Experimental data.

Number	Position (cm)	Successes	Average Time (s)	Average Success Rate
1	(30, 20, 45)	46	23.19	91.5%
2	(30, −20, 45)	45	18.71	
3	(30, 20, 15)	47	23.34	
4	(30, 20, 15)	44	22.49	
5	(50, −20, 45)	45	21.28	
6	(50, −20, 45)	46	22.30	
7	(50, 20, 15)	47	25.63	
8	(50, −20, 15)	46	25.07	

**Table 5:** Experimental data.

	Number	Color	Successes	Average Time (s)	Average Success Rate
a	1	red	47	18.10	94.5%
	2	red	48	15.15	
	3	green	0		
	4	red	49	19.36	
	5	red	45	23.11	
b	1	red	48	18.10	93.0%
	2	green	0		
	3	red	47	15.15	
	4	red	47	19.36	
	5	red	44	23.11	
c	1	red	42	18.10	90.0%
	2	red	47	15.15	
	3	red	48	23.11	
	4	red	43	19.36	
	5	green	0		
d	1	green	0		92.5%
	2	red	47	15.15	
	3	red	49	18.10	
	4	red	43	19.36	
	5	red	46	23.11	
e	1	red	47	22.14	92.4%
	2	red	44	20.47	
	3	red	48	23.46	
	4	red	47	18.65	
	5	red	45	21.22	
